# A novel adaptation of spatial interpolation methods to map health attitudes related to COVID-19

**DOI:** 10.1186/s12919-023-00264-z

**Published:** 2023-07-17

**Authors:** Raisa Behal, Kenneth Davis, Jeffrey Doering

**Affiliations:** 1 Fraym, 3101 Wilson Blvd., Suite 300, Arlington, VA 22201 USA; 2 Johnson and Johnson, 1 Johnson and Johnson Plaza, New Brunswick, NJ 08933 USA

**Keywords:** Machine Learning, Geospatial, Vaccine Hesitancy, Misinformation, Behavior Change, Immunization

## Abstract

**Background:**

The COVID-19 pandemic presented substantial challenges to public health stakeholders working to vaccinate populations against the disease, particularly among vaccine hesitant individuals in low- and middle-income countries. Data on the determinants of vaccine hesitancy are scarce, and often available only at the national level. In this paper, our goal is to inform programmatic decision making in support of local vaccine uptake. Our analytical objectives to support this goal are to (1) reliably estimate attitudinal data at the hyperlocal level, and (2) estimate the loss of data heterogeneity among these attitudinal indicators at higher levels of aggregation. With hyperlocal attitudinal data on the determinants of vaccine hesitancy, public health stakeholders can better tailor interventions aimed at increasing uptake sub-nationally, and even down to the individual vaccination site or neighborhood.

**Methods:**

We estimated attitudinal data on the determinants of vaccine hesitancy as framed by the WHO’s Confidence, Complacency, and Convenience (“3Cs”) Model of Vaccine Hesitancy using a nationally and regionally representative household survey of 4,922 adults aged 18 and above, collected in February 2022. This custom survey was designed to collect information on attitudes towards COVID-19 and concerns about the COVID-19 vaccine. A machine learning (ML) framework was used to spatially interpolate metrics representative of the 3Cs at a one square kilometer (1km^2^) resolution using approximately 130 spatial covariates from high-resolution satellite imagery, and 24 covariates from the 2018 Nigeria Demographic and Health Survey (DHS).

**Results:**

Spatial interpolated hyperlocal estimates of the 3Cs captured significant information on attitudes towards COVID-19 and COVID-19 vaccines. The interpolated estimates held increased heterogeneity within each subsequent level of disaggregation, with most variation at the 1km^2^ level.

**Conclusions:**

Our findings demonstrate that a) attitudinal data can be successfully estimated at the hyperlocal level, and b) the determinants of COVID-19 vaccine hesitancy have large spatial variance that cannot be captured through national surveys alone. Access to community level attitudes toward vaccine safety and efficacy; vaccination access, time, and financial burden; and COVID-19 beliefs and infection concerns presents novel implications for public health practitioners and policymakers seeking to increase COVID-19 vaccine uptake through more customized community-level interventions.

**Supplementary Information:**

The online version contains supplementary material available at 10.1186/s12919-023-00264-z.

## Background

As of late September 2022, over 6.5 million known individuals have died as a result of COVID-19 and over 12 billion COVID-19 vaccines have been administered [1]. During the first 12 months that the vaccine was available, vaccines yielded an estimated 14.4 million deaths averted, based on official COVID-19 mortality counts, or 19.8 million deaths averted using excess death figures across 185 countries globally [2]. Despite this, the majority of low-income countries have vaccination rates below 40% for target populations, compared to rates above 70% in most high-income countries [1].

### Need for a hyperlocal response

Several challenges have hampered vaccine uptake in low- and middle-income countries (LMICs), particularly in sub-Saharan Africa, including vaccine supply, attitudes towards vaccination [3], and last mile logistics. According to the Strategic Advisory Group of Experts on Immunization (SAGE) - the primary advisory committee to the World Health Organization (WHO) for immunization and vaccination - vaccine uptake is a complex and dynamic global problem with determinants that vary a) depending on context, time, place, program, and specific vaccine, b) across and within countries, and c) between population subgroups based on geographic, cultural, socioeconomic, and other factors [4]. For government health agencies and implementing organizations to be able to conduct effective vaccination programs within their respective locations, developing a local understanding of barriers to vaccine uptake as well as determinants of hesitancy is critical.

### Lack of sub-national data

Traditionally data on vaccine hesitancy is limited to available datasets that are typically representative of either the national, or at most the first administrative level [5]. National-level statistics regarding access and attitudes towards vaccines, while helpful, obscure local differences and are not informative in developing locally targeted vaccination programs, or risk communication and community engagement programs. However, identifying more granular variation in the determinants of vaccine hesitancy, which are driven by both population characteristics as well as attitudes, presents a challenge. Previous research has relied on non-representative samples supplemented by very large sample sizes collected through social media websites such as Facebook [6–8]. However, subsequent research has highlighted the limited utility of increasing sample size to overcome a non-representative sampling methodology [9].

### Benefits of spatial interpolation

Spatial interpolation methods driven by machine learning algorithms provide an avenue for estimating hyperlocal data that would otherwise be cost prohibitive. Collecting data at the hyperlocal level is usually restricted to large scale surveys like national censuses due to the time and resource intensity of data collection. Spatial interpolation methods allow us to instead impute hyperlocal data at a fraction of the cost due to smaller sample size requirements since these methods only require demographically and geographically representative household surveys and high-resolution satellite imagery to estimate data across missing clusters in the survey data. Spatial interpolation has been used previously [10], including in a public health context to understand patterns of HIV risk among adolescent girls and young women [11] and to create spatial layers using the Demographic and Health Surveys (DHS) [12].

Spatial interpolation methods were used by this project to create highly detailed, hyperlocal data at a 1km^2^ level to aid in understanding the geographic distribution of factors that might help or hinder vaccination efforts. This level of granularity was chosen for three key reasons: (1) many of the inputs to the modeling process are derived from high resolution satellite imagery that is more readily available at the 1km^2^ level; (2) this is sufficiently granular to inform community level public health programming, such as immunization microplanning; and (3) estimates can easily be aggregated to any custom catchment area or administrative boundary larger than 1km^2^. More information on how 1km^2^ level estimates readily align with existing Nigeria health microplanning can be found in the discussion section below. Of particular interest was the development of metrics that speak to health attitudes and behaviors, as machine-learning driven methods of spatial interpolation have not yet been utilized to interpolate representative attitudinal data at a hyperlocal level. This novel application of spatial interpolation methods has potentially wide-spread utility for public health practitioners in understanding behavioral drivers and barriers to public health interventions around the globe for a plethora of disease states and health issues beyond COVID-19.

### The 3Cs of vaccine hesitancy

The SAGE Working Group report highlights the utility of the Confidence, Complacency, and Convenience (3Cs) model of vaccine hesitancy, wherein the 3Cs are three primary determinants that either bolster or hinder vaccination against a given disease in a given location [4]. Confidence is an individual’s trust in the safety and efficacy of the vaccine, the system that delivers them, and the motivations of policymakers [4]. Complacency is an individual’s perceived risk to a disease and necessity of preventative action [4]. Convenience is the availability, affordability, accessibility, and appeal of immunization services [4]. These determinants, together, provided the necessary framework for understanding the underlying drivers and barriers to COVID-19 vaccine uptake, including attitudinal determinants. The 3Cs model also allows us to capture the heterogeneity of vaccine uptake determinants between subgroups across countries. For public health microplanners or decentralized health intervention resource management, hyperlocal data on attitudes towards the risk of COVID-19 and concerns about vaccination can support more efficient targeting of resources and more effective local program design.

### The multi-country project

Johnson and Johnson’s Global Public Health (J&J GPH) team partnered with Fraym, a producer of geospatial population data, as part of an initiative to support the uptake of all types of COVID-19 vaccines in sub-Saharan Africa. Fraym’s contribution was to develop hyperlocal data on population characteristics and attitudes related to COVID-19 across Ethiopia, Ghana, Kenya, Malawi, Mali, Nigeria, Rwanda, South Africa, Uganda, and Zambia. For several of these countries our project team used existing and publicly available data to develop ‘*proxy*’ models, which estimated the determinants of vaccine hesitancy Since these data were primarily collected prior to the COVID-19 pandemic, they lacked direct questioning on perceptions of COVID-19 and concerns regarding COVID-19 vaccination. Due to this limitation, proxy indicators were used to capture trends in expected behavior.

Over the last two years, our team conducted custom national and regionally representative household surveys in select countries to fill this gap in information, which allowed us to develop ‘*COVID-19*’ models using more explicit data on population attitudes around COVID-19 and vaccination. The COVID-19 model was applied in Ghana, Kenya, Nigeria, and South Africa. A detailed view of which model and data were used across countries is available below in Table 1. For the purpose of this paper, we focus on the application of the COVID-19 model in Nigeria to highlight the use of attitudinal data for hyperlocal targeting and microplanning.

**Table 1:** Data source summary^a^

Country	Data source	Additional data sources
***Proxy models***^***b***^		
*Ethiopia*	Demographic and Health Survey 2016	Afrobarometer 2021
*Malawi*	Demographic and Health Survey 2015–16	Afrobarometer 2020
*Mali*	Demographic and Health Survey 2018	Afrobarometer 2019
*Nigeria*	Demographic and Health Survey 2018	Afrobarometer 2021
*Rwanda*^*c*^	Demographic and Health Survey 2019–20	
*Uganda*	Demographic and Health Survey 2016	Afrobarometer 2019
*Zambia*	Demographic and Health Survey 2018	Afrobarometer 2017
***COVID-19 models***		
*Ghana*	Fraym 2022	
*Kenya*^*d*^	Fraym 2021	
	Fraym 2022	
*Nigeria*	Fraym 2022	
*South Africa*	Fraym 2021	

^a^This study and manuscript only used the Fraym 2022 Nigeria survey. All other data sources and countries referenced in this table are provided only for additional context

^b^The proxy models developed convenience and complacency indices using proxy indicators from the Demographic and Health Surveys, and confidence indices using indicators on trust in government actors and institutions from the Afrobarometer Surveys

^c^Proxy data for the confidence index was not available in Rwanda

^d^Two rounds of data were collected in Kenya, resulting in two separate results

## Methods

In the Methods section we begin with an overview of the multi-step process the project team developed to adapt the WHO’s 3Cs framework to the given country and data source, followed by an explanation of the spatial interpolation process adapted for attitudinal data. Finally, we cover the specifications of the COVID-19 model for Nigeria including selected indicators.

### An overview: adapting the WHO’s 3Cs model of vaccine hesitancy

The WHO’s 3Cs framework defined by the SAGE Working Group informed the development of a multi-step model to map the determinants of vaccine hesitancy, namely confidence, complacency, and convenience. As mentioned above, two models, the proxy model and the COVID-19 model, were developed to track these determinants to respond to varying data availability across countries. Below, we provide an overview of the customizable process we developed to model and map each of the 3Cs using either data input.

The 3Cs model development has three broad steps. In the first step, indicators from nationally representative and geo-referenced household survey data are mapped to each of the determinants of vaccine hesitancy such that the drivers of confidence, complacency, and convenience are well represented. These indicators are selected from household survey data, and as such the unit of analysis is the individual. These are considered the inputs to the model.

In the second step, an index is created for each determinant using Multiple Correspondence Analysis (MCA) so that confidence, complacency, and convenience are each individually represented by a single metric instead of several variables. Widely considered a counterpart to Principal Component Analysis (PCA), MCA is similarly a multivariate statistical method to represent underlying characteristics, but of unilaterally categorical data [13]. Since the data identified in step one is largely categorical, any remaining mapped inputs that are continuous are transformed to categorical variables. We confirm the directionality of inputs to match the direction of the index such that, for example, all inputs in the convenience model increase in the direction of higher convenience. We subsequently created a single MCA index to represent each of the 3Cs. Like the inputs, the final indices are also created at the individual level in the survey data, so that we can track variation in confidence, complacency, and convenience across individual respondents.

The construction of the convenience index differs slightly from that of confidence and complacency. The confidence and complacency indices are each developed using all respective inputs in a single index. The convenience index on the other hand is developed as a composite index. Convenience is an amalgamation of several distinct barriers to vaccine uptake, since one may face either accessibility, time, or financial burdens in seeking health care. Respective to context, the latent drivers for each of these burdens may be unrelated and should therefore be indexed separately. As such, the convenience index is the summation of two separate indices: the accessibility index and the time and financial burden index. Each final 3Cs index was normalized so they could be represented as a score ranging between zero and one hundred for easier interpretation, which also allows comparison across the indices.

To enable as much comparability as possible across the project countries, the index inputs were kept consistent as much as possible subject to data availability, respective to the model type. As such, *proxy* models across countries share the same input variables as collected by the respective DHS and Afrobarometer, while the *COVID-19* models share the same inputs as surveyed by the project team.

### Spatial interpolation

In the final step of the process the 3Cs indices are spatially interpolated to produce hyperlocal estimates. Spatial interpolation is enabled by an ML algorithm that processes various data, such as cluster-level survey data, high-resolution satellite imagery, and other derived data products that include earth observation and human settlement data and applies a spatial ensemble model to predict the missing survey data across the non-enumerated areas [11]. The final product is a geospatial raster layer where each pixel, represented here as a 1km^2^ grid, has an associated estimated value.

The spatial interpolation process also includes steps to ensure the validity of the model estimation. The interpolation algorithm includes several stages of cross-validation of training and testing data across several statistical models, which validates grid level estimations, before selecting the final model(s) with the lowest root mean squared error (RMSE). Additionally, the RMSE of the null model, which includes all covariates, is confirmed to be higher than the final ML model RMSE to ensure efficient covariate selection. The next stage of validation confirms robustness of national or sub-national estimations and consists of two steps: calculating the absolute difference between the survey mean and the interpolated raster mean at the level of representativeness of the base survey and identifying the statistical significance of the absolute difference by referencing the confidence intervals of the survey data. Additional checks, such as mean comparison of lower levels of administrative divisions and checking survey cluster distributions are reserved if the layer does not meet the primary validation criteria. A more detailed explanation of the interpolation process is provided in Additional file 1: Appendix A.

To interpolate attitudinal data, updates were made to the spatial interpolation ML model to ensure model performance. Based on a literature review of the leading attitudinal indicator, in this example vaccine hesitancy in Nigeria, we identified additional features to be included as inputs in the ML model. We identified several demographic and socioeconomic indicators that were associated with attitudes around vaccination in Nigeria. Some examples include gender [14], religion [15], and wealth [16]. These indicators were pulled from the Nigeria Demographic and Health Survey 2018 [17] and were first interpolated using our base interpolation process. The output layers served as inputs in the interpolation of the 3Cs indices and any other indicator capturing an opinion or attitude.

### Applying the 3Cs model to Nigeria

#### Household survey

To develop the COVID-19 model, a nationally representative and georeferenced survey was developed by Fraym and administered by an external firm, GeoPoll, in February 2022. The survey was administered as a Computer Assisted Telephonic Interview (CATI) across 4,922 respondents aged 18 and above. The interviews were conducted in English, Hausa, Yoruba, and Igbo.

The survey itself included questions on demographic and socioeconomic characteristics, media consumption, plans to receive the COVID-19 vaccine, and attitudes towards COVID-19 and the COVID-19 vaccine. The survey instrument contained separate sections that asked concerns about accessing the vaccine, perceived risk of the virus, and confidence in the safety and efficacy of the vaccine. These sections were used to develop the confidence, complacency, and convenience indices, respectively.

To improve representativeness of a phone survey, the sample design included both demographic and socioeconomic quotas for sample selection, and the final data were weighted post-processing. The demographic quotas were based on data from the U.S. Census Bureau’s PEPFAR program and were nested by geopolitical zone, age, and sex [18]. Whereas the socioeconomic quotas were based on national rates of bank account ownership and connection to the electrical grid as per the 2018 DHS due to the strength of their relationship with the DHS wealth index. The collected and processed data were finally weighted using an Iterative Proportional Fitting (IPF) algorithm that matched the frequencies of the survey data to reference data, which were nationally representative. The scaling factors used were urbanicity and gender from the United Nations 2022 Revision of World Population Prospects report, and the same socioeconomic asset distribution used for the sample quotas from the DHS [19]. The IPF process achieved convergence within three iterations and prevalence rates were confirmed at the first administrative level.

#### Selected indicators

To track confidence in the COVID-19 vaccine, we asked respondents about their attitudes toward the safety and efficacy of the vaccine, as well as the vaccination status of people they trust. Vaccine complacency was captured in terms of perceived personal and community risk from COVID-19, via questions regarding belief that COVID-19 is not real, or belief that being healthy precludes concerns about hospitalization. To measure vaccine convenience, we tracked concerns related to accessibility, time, and financial burdens that were associated with receiving the vaccine. To better capture accessibility burdens, we included external data on distance to medium and large health facilities. The location of these health facilities in Nigeria was accessed via a WHO list of health facilities sourced from several government and non-government sources from 50 countries in Sub-Saharan Africa [20]. Spatial friction layers accessed via the Malaria Atlas Project allowed us to estimate least cost walking and driving time to these health facilities which account for travel costs such as slope and terrain [21]. The complete list of inputs can be found in Table 2 below. Our rationale for choosing the indicators and questions below, as well as for aligning them to the 3Cs model, is detailed further in Additional file 2: Appendix B.

**Table 2:** Selected inputs per 3Cs index

**Confidence index**	**Convenience index**		**Complacency index**
***Concerns about receiving the COVID-19 vaccine***	***Concerns about the ability to access the COVID-19 vaccine***		***Feelings about the spread of COVID-19***
COVID-19 vaccines may not be safe	*Accessibility index*	Walking time to WHO medium and large health facilities^a^	My friends and family are not at risk of COVID-19
COVID-19 vaccines may not be effective		Driving time to WHO medium and large health facilities^a^	I am healthy and do not need to worry about being hospitalized
The COVID-19 vaccine will not be effective against new strands		Individual lives in a household that owns a scooter	COVID-19 is not spreading in my community
People I trust are not getting vaccinated	*Time and financial burden index*	There will not be enough COVID-19 vaccines	COVID-19 is not real
		Do not know where to get a COVID-19 vaccine	I already had COVID-19 and am not afraid I will spread it
		Will not have time to get the COVID-19 vaccine	
		There may be a financial cost associated	

^a^Distance to WHO medium and large health facilities was not reported by survey respondents. Distances were calculated using geo-coordinates for survey respondents and WHO health facilities, and friction layers from the Malaria Atlas Project

#### 3Cs index terciles

Due to the nature of their construction, the 3Cs indices each reflect a relative score of prevalent concerns. Although the score allows us to compare levels of an index across hyperlocal communities, it does not yield itself to actionable insights on specific populations. As such, prior to interpolation when the indices were constructed using individual level survey data, the indices underwent an additional step where they were spliced into separate quantiles each representing a different level of the determinant. In most cases, including Nigeria, we were able to achieve a maximum of three quantiles, also referred to as terciles. Although there were some cases in other project countries where lack of 3C variation only afforded us 2 quantiles. As such, in the survey data we classified not only the confidence index for an individual, but also whether they were relatively low, medium, or high confidence. These terciles were also interpolated to reveal the proportion of the population who are low confidence at the hyperlocal, state, or national level. Combining these data on population proportions with hyperlocal estimates of population density from WorldPop [22], we can also map the total number of people who are low confidence at various geographic levels. This allows implementers to design programs around the proportion as well as number of people in a community or LGA who have low confidence.

### Additional data for hyperlocal targeting

In addition to interpolating the 3Cs indices and their respective terciles, we also interpolated several population characteristics from the household survey, which included demographics, socioeconomics, and media usage. We were also able to identify the locations of public and private COVID-19 vaccination sites using data from The National Primary Health Care Development Agency, a parastatal of the Federal Ministry of Health [23]. These geo-referenced vaccination sites were overlaid on mapped output, but not directly included in index construction or manipulated for analysis. This data can be used to design targeted interventions and media campaigns at the hyperlocal level. Examples of how to use these data in conjunction with the 3Cs are shared in the Discussion section.

### Analysis plan

In support of our dual analytical objectives, our statistical analysis plan is disaggregated into the following steps. To verify the reliable estimation of attitudinal data at the hyperlocal level, we confirm the sufficient capture of attitudes in the 3Cs indices and validate the results of the spatial interpolation. We also test the operationalization of the interpolations, such as by identifying the prevalent determinant at the hyperlocal level. This analysis is shared in *Results*. To better understand the heterogeneity of population characteristics and indicators of interest at the hyperlocal level, and the potential loss of information from increasing levels of aggregation, we explore three examples of hyperlocal variance in population characteristics which can be used for more impactful decision making by local implementers. These examples are shared in the *Discussion*.

## Results

In this section we prioritize our first analytical objective, which is to reliably estimate attitudinal data at the hyperlocal level. We first explore the MCA indices constructed for each of the 3Cs, and the primary drivers of each. This is followed by a summary of the performance and results of the spatial interpolation models of vaccination attitudes.

### MCA performance

The Confidence, Complacency, and Convenience (3Cs) MCA indices were each constructed and verified across their inputs to ensure input value addition and expected direction of correlation between the inputs and the respective index. The primary dimensions of all the MCA indices of attitudinal inputs, barring one, surpassed a minimum of 90 percent explained variance. Only the accessibility index, which contained the walking and driving times to health facilities and scooter ownership, achieved a primary dimension that explained 74 percent of variation.

### Drivers of the determinants

Since the 3Cs scores are constructed as MCA indices and are composites of survey inputs (Table 2), we can test the pairwise correlations between each index input and the index itself to determine the contribution of inputs to the larger whole (Table 3). Understanding the drivers of determinants can shed light on the most pressing concerns of the population, especially since they can vary across countries and across time as attitudes and priorities change. Identifying these drivers can inform program design by streamlining the focus of interventions.

**Table 3:** Correlations of 3Cs indices and their inputs

**Confidence index**		**Convenience index**		**Complacency index**	
***Concerns about receiving the COVID-19 vaccine***		***Concerns about the ability to access the COVID-19 vaccine***		***Feelings about the spread of COVID-19 vaccine***	
COVID-19 vaccines may not be safe	-0.73*	Walking time to WHO medium and large health facilities^a^	-0.67*	My friends and family are not at risk of COVID-19	0.70*
COVID-19 vaccines may not be effective	-0.73*	Driving time to WHO medium and large health facilities^a^	-0.65*	I am healthy and do not need to worry about being hospitalized	0.67*
The COVID-19 vaccine will not be effective against new strands	-0.65*	There will not be enough COVID-19 vaccines	-0.50*	COVID-19 is not spreading in my community	0.60*
People I trust are not getting vaccinated	-0.60*	Do not know where to get a COVID-19 vaccine	-0.46*	COVID-19 is not real	0.50*
		Will not have time to get the COVID-19 vaccine	-0.46*	I already had COVID19 and am not afraid I will spread it	0.48*
		There may be a financial cost associated	-0.46*		

^*^Correlations that are statistically significant at the 95% confidence level

^a^Distance to WHO medium and large health facilities was not reported by survey respondents. Distances were calculated using geo-coordinates for survey respondents and WHO health facilities, and friction layers from the Malaria Atlas Project

### Spatial interpolation model performance

Upon interpolation, the 3Cs index layers underwent standard data quality checks (as described in the Methods section). The final ML models selected for spatial interpolation of the 3Cs indices and respective terciles yielded a root mean squared error below 0.05. Since the survey collected for this project was representative at the first administrative level, i.e., states, we compared the survey and raster means across the thirty-six states of Nigeria. Although the confidence and complacency index interpolations largely passed our validation criteria, the convenience index layer had lower model performance across six states: Adamawa, Akwa Ibom, Bayelsa, Enugu, Gombe, and Nasarawa. As such we advise that caution be applied to estimates from these states.

### Interpolation results

**Fig. 1:**
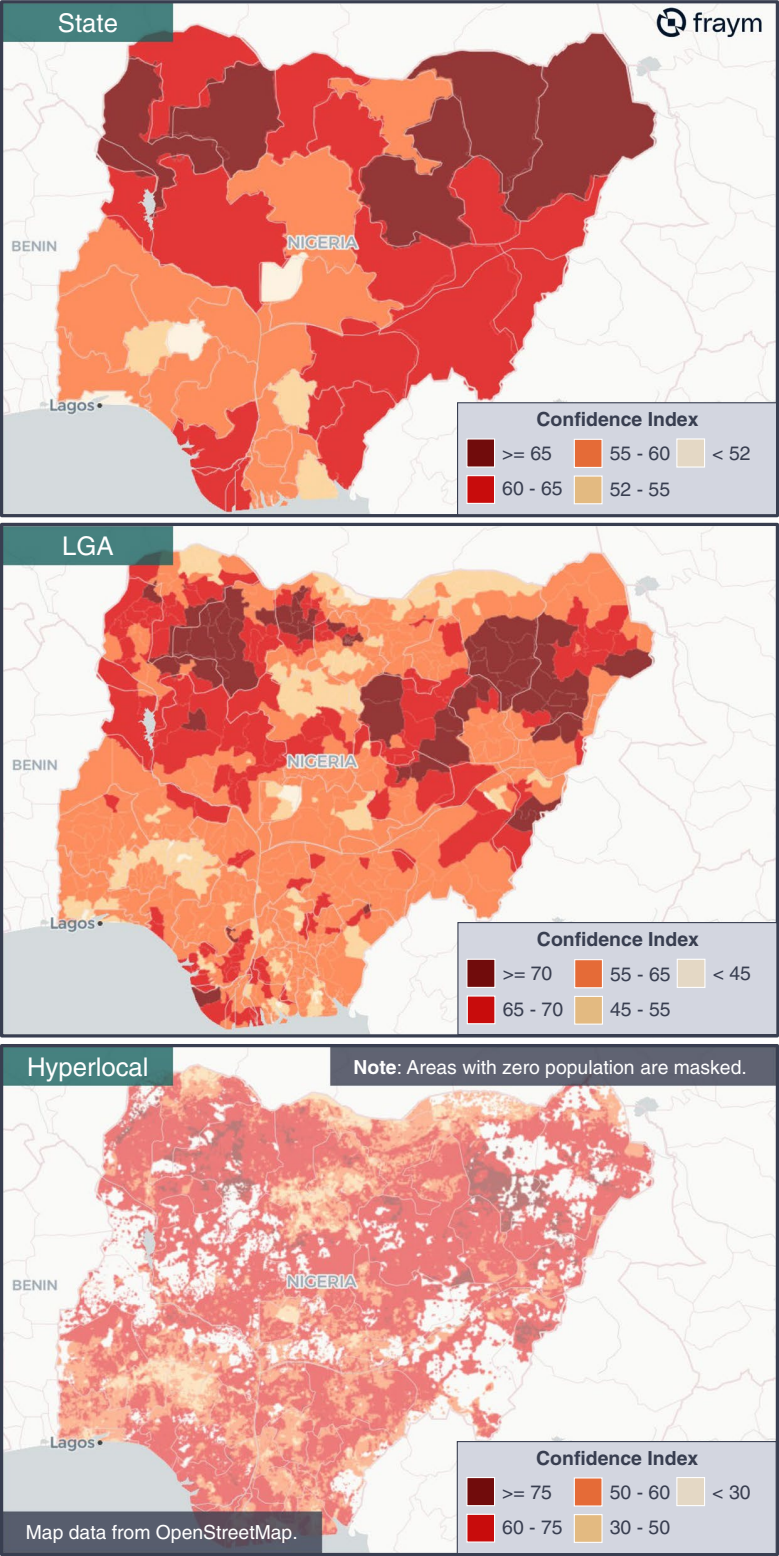
Mapping the confidence index

Spatial layers for each of the 3Cs and their respective terciles were created at three geographic levels: the state and local government area (LGA) administrative divisions, as well as the hyperlocal (1km^2^) level. An example of the three views of the confidence index at various levels of granularity is shared in Fig. 1 below.

We also produced national averages of the 3Cs indices and terciles to compare our subnational data against. We found that these national averages obscure the significant subnational heterogeneity of the 3Cs. For example, at the national level 30% of individuals in Nigeria were “low confidence” with a national score of 59 on the confidence index. Across LGAs, the average proportion of individuals with “low confidence” ranged from 8% all the way up to 60% while the average scores of the confidence index ranged from 36 all the way to 80. This variance increases further at the hyperlocal level as Table 4 demonstrates below.

**Table 4:** Summary of interpolations: 3Cs indices and respective levels

**Average score across 3Cs indices**	**Confidence index**	**Complacency index**	**Convenience index**
*Nigeria*	59	51	30
*State (range)*	48—69	41—63	19—49
*LGA (range)*	36—80	27—70	13—64
*Hyperlocal (range)*	12—93	8—80	0—78
**Average rate across 3Cs levels**	**Low confidence**	**High complacency**	**Low convenience**
*Nigeria*	30%	37%	84%
*State (range)*	17%—40%	19%—50%	65%—93%
*LGA (range)*	8%—60%	10%—70%	42%—100%
*Hyperlocal (range)*	0%—93%	0%—91%	15—100

In addition to subnational heterogeneity, we also investigated intra-state heterogeneity to better understand the level of variance within a given state, especially between LGAs and hyperlocal 1km^2^ grids. To simplify our analysis across the 3Cs indices, we created a Mean Cumulative Score (MCS) to represent the average range across the 3Cs. We can now compare the MCS across states, and between LGAs and hyperlocal grids. For example, we find that Katsina has the highest (30 MCS) and Bayelsa the lowest (10 MCS) mean heterogeneity at the LGA level. Despite this, Bayelsa has significant *intra-LGA* heterogeneity, i.e., the hyperlocal grid level (a 34-point range in confidence, 26-point range in complacency, 53-point range in convenience, or 38 MCS). Interestingly the average range in heterogeneity for Bayelsa at the *hyperlocal level* (38 MCS) is higher even than the state with the most heterogeneity at the *LGA level* (Katsina state, 30 MCS).

This suggests that the significant amount of hyperlocal heterogeneity found in each 3C index across Nigeria (as presented above in Table 4) is likely due to both inter- and intra-state heterogeneity. Table 5 below summarizes intra-state heterogeneity for both Katsina and Bayelsa states at the LGA and hyperlocal range.

**Table 5:** Highest and lowest intra-state heterogeneity across 3Cs indices

	**Confidence index**	**Complacency index**	**Convenience index**	**Cumulative score**	**Mean cumulative score**
*Katsina State (LGA range)*	38 – 76 (38)	40 – 60 (20)	53 – 20 (33)	91	30
*Katsina State (hyperlocal range)*	28 – 81 (53)	32 – 79 (47)	2 – 68 (66)	166	55
*Bayelsa State (LGA range)*	58 – 70 (12)	37 – 44 (7)	15 – 27 (12)	31	10
*Bayelsa State (hyperlocal range)*	50 – 84 (34)	28 – 54 (26)	0 – 53 (53)	113	38

### Prevalent determinant of vaccine hesitancy at the hyperlocal level

**Fig. 2:**
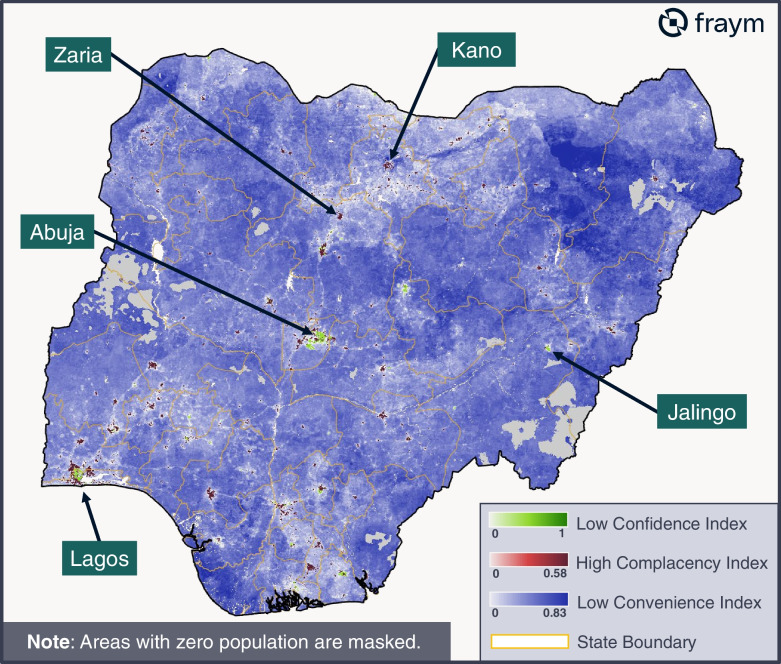
Heatmap of prevalent determinant of vaccine hesitancy at the hyperlocal level

In addition to mapping each individual 3Cs determinant, we mapped a community’s prevalent 3Cs determinant, defined as the index with the highest score per grid cell., This required inverting the confidence and convenience indices so that higher scores reflect a *decrease* in confidence and convenience, respectively. These are paired with the complacency index, where higher scores reflect an *increase* in complacency attitudes. As seen in Fig. 2 below, the prevalent determinant of vaccine hesitancy for communities across Nigeria is low levels of convenience. The exception is that in urban centers, higher levels of complacency are more prevalent. This is likely related to urban centers typically having greater access to vaccination centers and therefore higher convenience scores. Low rates of confidence are also evident in Abuja and Lagos.

### Population characteristics for hyperlocal targeting

Sociodemographic characteristics and media-usage patterns were also interpolated to demonstrate the use of hyperlocal data to target populations according to the prevalent or significant determinants of vaccine hesitancy. A sample of these population characteristics are shared for Lagos in Example 2 in the Discussion section below.

## Discussion

Our first analytical objective for this study was to reliably estimate attitudinal data, specifically concerning vaccine hesitancy, at the hyperlocal level. The previous section makes the case that we can successfully build informative metrics to capture vaccine hesitancy and spatially interpolate these data at the 1km^2^ level. In this section, we would like to address our second objective which is to explore the loss of heterogeneity in our indicators of interest at higher levels of aggregation and discuss the ways in which public health stakeholders can best utilize hyperlocal data for targeted program design and implementation.

### Benefits of hyperlocal data

The merits of hyperlocal estimates are evident in the variance of estimates captured at lower geographic levels of data. As noted in Table 4 the 3Cs indices produced at the state level had a range of approximately 24 points on average, whereas our hyperlocal estimates provided a range of approximately 77 points. This can be interpreted as a 52-point loss in variance by using state level data instead of hyperlocal. The same is true when viewing the 3Cs index tercile estimates. Public health stakeholders using traditional models of tracking determinants of vaccine hesitancy at a national level, or even at the first administrative division, are making decisions with imprecise and imperfect information that disguises the true heterogeneity in population characteristics and attitudes.

Hyperlocal estimates, especially at the 1km^2^ level, are increasingly prevalent within existing public health planning and programming and are already being used to inform microplanning for immunization in many countries including Nigeria. Most notable has been a growing interest in microplanning at the health facility level to improve targeting and programming in respective catchment areas. Data at the 1km^2^ level is optimal for constructing custom catchment areas. As far back as 2009, the WHO has issued guidance outlining the utility of developing microplans at the health facility level using catchment area analysis to identify hard to reach locations and populations in efforts to increase immunization rates [24]. Since 2017 the WHO Reaching Every District (RED) strategy, though national in scope, has been implemented at the district and health facility levels [25]. And in 2021, the WHO published updated guidance on operational microplanning, specifically for COVID-19 vaccination [26]. In Nigeria specifically, a study of the utility of digital microplanning using geographic information systems (GIS) also used health clinic catchment areas to align with government requirements, which included service distances of 0-2 km, 2-5 km, and 5-10 km [27]. The 1km^2^ level data produced by this project can be used explicitly and directly to inform the recommended health facility catchment area analysis for effective microplanning, especially in Nigeria where the government’s service distances are factors of 1km^2^. Even beyond the 1km^2^, our hyperlocal data can be aggregated to the district level (LGA in Nigeria) which is still much more granular than standard survey estimates, which are representative only at the national or first administrative level, such as the Nigeria 2018 DHS.

Below we share three examples of how our hyperlocal estimates can be used to provide far more granular, nuanced insights than survey data alone.

#### Example 1: Merits of all 3 convenience indices

The availability of data on COVID-19 vaccination sites from the Federal Ministry of Health in conjunction with the convenience index allowed for an analysis of barriers faced by communities in proximity to vaccinations sites. Contrary to expectation, we found that proximity to a COVID-19 vaccination site was not always a strong predictor of a high convenience score within a community. This disparity was often due to the very different underlying drivers of convenience across accessibility, time, and financial burdens of receiving vaccination. Since the convenience index is a composite of two distinct indices, we can separate convenience concerns across physical accessibility and more subjective perceived time and financial burdens of seeking the vaccine. We found that communities with low travel times to a vaccination center could still have low convenience if they were more likely to be concerned about the time and cost of receiving the vaccine.

**Fig. 3:**
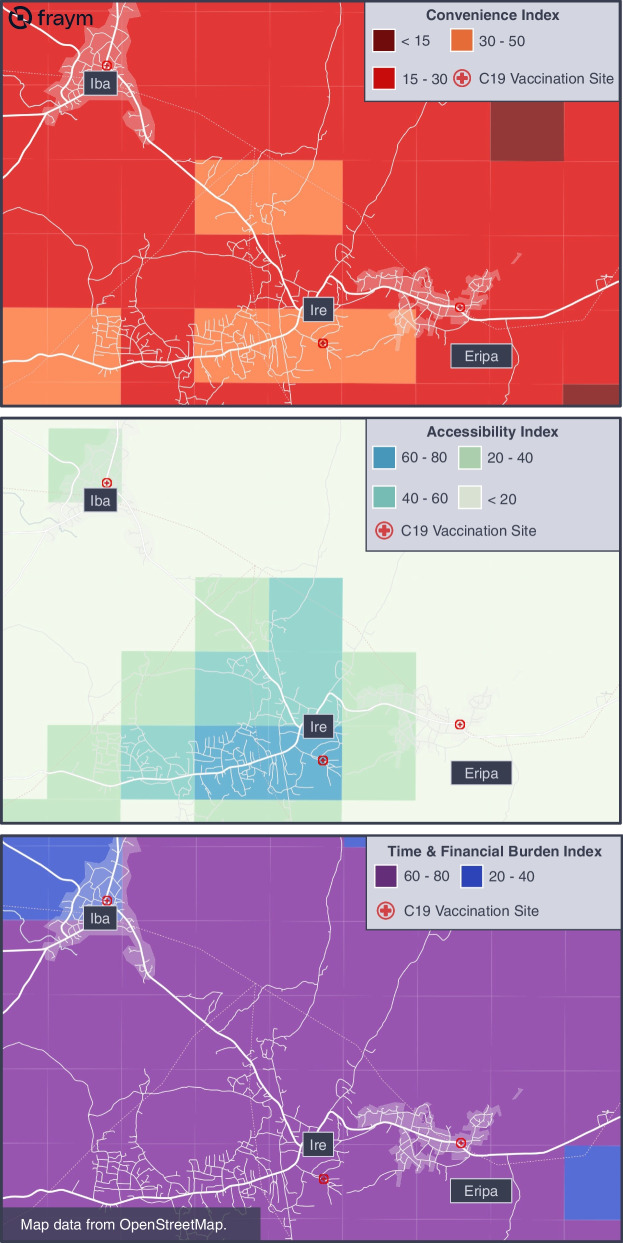
Heatmaps of convenience, accessibility, and time and financial burden indices in Osun State

For example, although the towns of Iba, Ire, and Eripa in the state of Osun (Fig. 3) are each served by the three COVID-19 vaccination sites, none of the neighborhoods had a convenience index greater than 50, with most of them falling between 15–30 (out of 100). That is, despite high physical proximity to vaccination sites other factors were pushing down their convenience score, such as high scores on the time and financial burden index, with most neighborhoods falling between 70–85.

The maps above highlight three important findings: (1) convenience can be a significant barrier to vaccination even in communities with COVID-19 vaccination sites, (2) there can be significant heterogeneity in convenience in neighborhoods served by the same vaccination site, and (3) the underlying components of convenience can point in very different directions, so should ideally be investigated concurrently.

#### Example 2: Profile of high complacency population

**Fig. 4:**
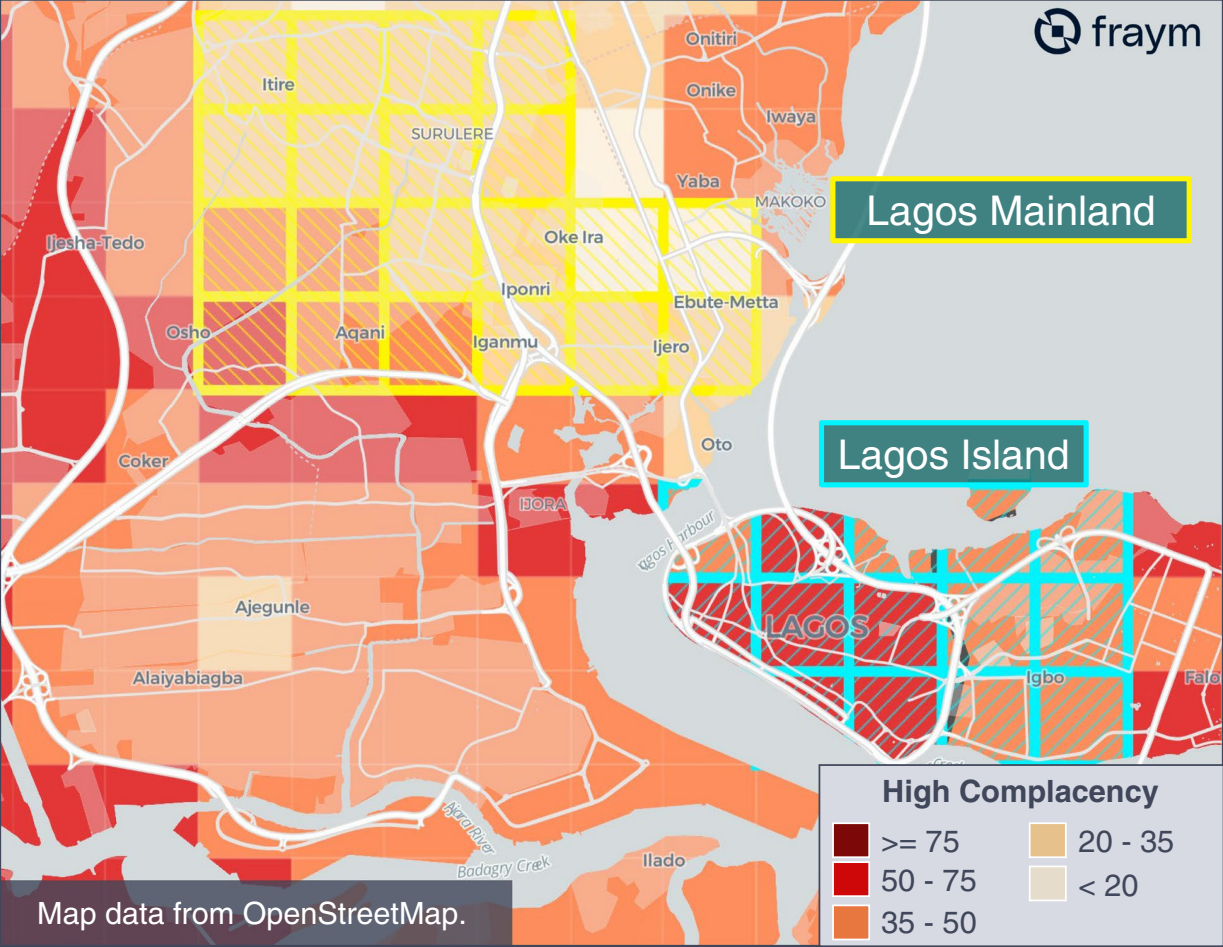
Heatmap of high complacency population (%) in Lagos, Nigeria

In the example below, we highlight population heterogeneity across Lagos state to emphasize (a) variation in complacency rates across adjacent neighborhoods, and (b) variation in characteristics across high complacency populations. We first selected two communities across adjacent LGAs in urban Lagos: Lagos Mainland and Lagos Island (Fig. 4). Although located right next to each other, just 29% of the population in the selected Lagos Mainland neighborhoods have high complacency, compared to 51% in Lagos Island. For reference, the average rate of high complacency individuals is 40% across Lagos state. As seen in Fig. 4, this variation is evident across selected neighborhoods within respective LGAs. This highlights how nearby neighborhoods can have very different opinions about their likelihood of catching, spreading, or experiencing negative health outcomes from COVID-19 and require different messaging campaigns.

Further study of each neighborhood’s population characteristics revealed other significant differences. Compared to Lagos Mainland, the more densely populated Lagos Island neighborhoods were less likely to use Facebook or own a radio and more likely to be Catholic and watch television programming on *Arewa 24*. These and additional characteristics on media usage can be viewed below in Table 6 which contains sample averages of neighborhoods in Lagos Island and Lagos Mainland, as well as the respective difference in means.

**Table 6:** Summary statistics of population characteristics in Lagos, Nigeria

Indicator	Lagos Island	Lagos Mainland	Difference^a^
High complacency	51%	29%	22%
***Demographics***			
*Population*	176,030	527,430	
*Ages 17 and under*	32%	36%	4%
*Ages 18–24*	17%	16%	1%
*Ages 25–34*	22%	20%	2%
*Ages 35–44*	13%	13%	1%
*Ages 45 and over*	16%	15%	0%
*Muslim*	15%	21%	6%
*Protestant*	63%	68%	6%
*Catholic*	22%	11%	12%
***Top news sources***			
*Facebook users*	68%	82%	14%
*Twitter users*	32%	26%	6%
*YouTube users*	18%	29%	10%
*WhatsApp users*	78%	82%	6%
*Cool FM listeners*	45%	42%	2%
*Rhythm FM listeners*	35%	13%	22%
*Wazobia FM listeners*	42%	37%	5%
*TVC News viewers*	38%	47%	9%
*Arewa 24 viewers*	34%	16%	18%
*Channels TV viewers*	49%	49%	1%
*The Punch readers*	31%	26%	5%
*Vanguard readers*	34%	24%	10%
*Nigerian Tribune readers*	16%	23%	6%
***Household communication assets owned***			
*Radio ownership*	85%	96%	11%
*Television ownership*	93%	93%	1%
*Computer ownership*	17%	18%	0%
*Smartphone ownership*	97%	99%	2%

^a^Absolute difference in means

These differences have significant implications for tailoring both the message and the medium of public health communications or behavior change interventions. For example, messaging aimed at reducing vaccine complacency via television programming on *Arewa 24* would likely be more effective in the Lagos Island neighborhoods than the Lagos mainland neighborhoods.

#### Example 3: Designing a media campaign targeting low confidence populations

One of the more common tools for social behavior change to improve health outcomes is tailored media programming delivered via social media [28], television [29], radio [30], newspaper [31], and even community theater [32]. However, it would be impractical and inefficient to deploy a national level campaign across all major media channels, with media content simultaneously addressing vaccine confidence, complacency, and convenience related issues. Tailored content that is designed to address the determinants of vaccine hesitancy within specific subgroups [33], delivered via the channels they are most likely to receive, and provided in the language they speak at home, will likely utilize resources more efficiently and be more effective at increasing vaccine uptake.

For this reason, we have also provided hyperlocal estimates on several complementary indicators, including the proportion of users among fifteen different social media channels and applications, thirteen different television channels, twelve different radio stations, and eight different newspapers across Nigeria. Paired with the proportion of individuals that speak four different languages at home, the medium of messaging can now be tailored to an unprecedented level.

**Fig. 5:**
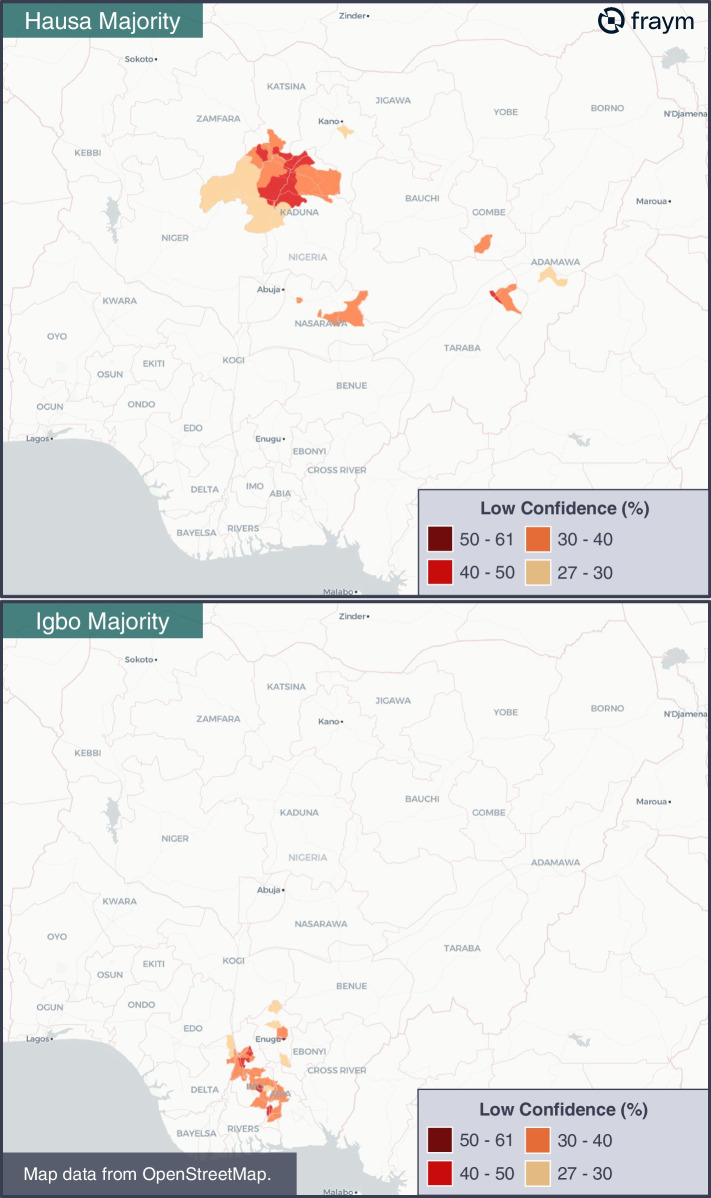
LGAs best suited for counter-misinformation content in Hausa and Igbo

Using the hyperlocal data on the 3Cs determinants of vaccine hesitancy, language, and media consumption, a public health stakeholder can design a custom media campaign to address low confidence among Hausa speakers across target LGAs. For example, they can devise media content to address medical misinformation regarding vaccine safety, deploy the media campaign in Hausa, and explicitly target smartphone users via Facebook Ads. To do so, we can isolate LGAs with at least 50% native Hausa speakers, that also have majority proportions^1^ of low confidence individuals^2^ smartphone owners^3^ and Facebook users^4^ (Fig. 5).

With these parameters, of the 774 LGAs the media campaign would be deployed in 17 of them across central Nigeria. If we wanted to run the same campaign but target Igbo speakers, we could refine the parameters to include only LGAs with at least 50% native Igbo speakers highlighting 45 separate LGAs across southern Nigeria to deploy the media campaign (Fig. 5). The value of hyperlocal data is evident in the ability to adjust resource allocation and targeting parameters rapidly and carefully for interventions aimed at increasing vaccine uptake.

### Study limitations

This section describes potential study and analytical limitations that we would like to note with respect to representativeness and generalizability of both the data we used as well as the data we estimated. We also comment on the limitations of our analytical objectives, which precluded more expansive analyses, but which could serve as avenues for future research. For each challenge, we also describe steps we have taken to mitigate it.

#### Representativeness of a phone survey

Data for this study was collected by means of phone surveys, which restricted the sampling frame to phone owning populations. However, we do not think this compromised the generalizability of our survey results due the prevalence of phone ownership in Nigeria as well as the survey specifications. As per the DHS, the rate of mobile phone ownership across households in 2018 was roughly 90%, and although male, urban, and richer households were more likely to have a mobile phone, this increased likelihood was marginal and unlikely to skew the distribution.

Moreover, to mitigate any loss of representativeness, the project (a) administered sample quotas for socioeconomic status and nested quotas for age, gender, and state and (b) implemented post-survey reweighting, specifically of primary factors such as age, gender, socioeconomic factors, and urbanicity. These steps are elaborated upon in the Methods section above. As such we believe that sufficient steps were taken to be able to interpolate primary indicators of this research related to COVID-19 vaccine hesitancy and maintain relevance for the general population.

#### Unrecognized data on vaccination sites

Lack of exhaustive data on COVID-19 vaccination sites, which may have been more plentiful than captured, can contribute to an underestimation of the accessibility index, and therefore also the convenience index. It can also confound the relationship with the perceived time and financial burden index since we currently observe populations that report low levels of physical proximity, but also high levels of perceived convenience. However, data on pop up clinics, roaming community vaccination drives, or other temporary or uncaptured vaccination options were unavailable for our analysis. Moreover, due to their temporary nature, knowledge of them can be less widespread than permanent health facilities and therefore have a less powerful relationship with perceived and overall convenience.

#### The uniform 3Cs model approach

The multi-national scope of this project led to prioritizing comparability of the 3Cs model across countries rather than customizing each to the local or national context. As discussed in Methods, index inputs varied between the input data sources i.e., DHS and the COVID-19 survey, and resulted in two different models, the Proxy model and COVID-19 Model, respectively. Although, within each model, the inputs were consistent across countries. In this manner, we could compare relative 3Cs indices scores between Nigeria and Kenya.

On the other hand, a limitation is that a country model was not customized or tested for internal validity. This leads to certain challenges, such as the use of scooter ownership as an input in the accessibility index, and thereby the convenience Index. This indicator was included across project countries as a measure of mobility or travel accessibility to a vaccination site even though, in Nigeria scooters are not typically used for private transportation. We can note though that this input was not a statistically significant driver of the final convenience index.

#### A cost–benefit analysis of hyperlocal targeting

A comparative analysis of the costs and benefits of using standard aggregated household data as opposed to hyperlocal data as put forth by this paper would be incredibly informative. However, this analysis was out of scope for this paper where our objectives were to test spatial interpolation of attitudinal data, and better understand the benefit of hyperlocal heterogeneity for programmatic decision making. We do believe that it would be a fascinating avenue for future research.

Prior to this study, we have observed that the cost–benefit trade-off in collecting and generating hyperlocal data is becoming increasingly favorable for large-scale, well-funded public health programs. This is primarily because data collection and generation costs using spatial interpolation and machine learning methods are not particularly sensitive to changes in health program scale or structure but remain fairly static. Many multi-year, multi-million USD donor-funded public health programs are already contributing significant resources to baseline, midline, and endline evaluations. In addition, as outlined in our research, these hyperlocal estimates provide community level insights across the entire country. With great inter- and intra-donor funded project coordination, nationally representative and georeferenced survey data with sufficient sample sizes to inform hyperlocal estimates could be afforded with a negligible impact on program cost. There is also an opportunity to use hyperlocal estimates to more efficiently and effectively allocate public health resources. Thus, the cost–benefit ratio derived from increasingly precise health programming and resource allocation will become increasingly beneficial for larger scale public health programs.

#### Heterogeneity within survey clusters

There are expected cases where an indicator of interest may vary within a single high-density urban cluster. For example, a high-density urban cluster may include some rich households and some poor living alongside each other. In such cases, the effect of the respective data points (or households) within this cluster may average into one another. The statistical contribution of that cluster overall will be lowered since its predictive power of the indicator of interest, such as socioeconomic status, decreases.

However, this ‘averaged’ cluster affects all grid estimates equally since the spatial interpolation model does not estimate a grid’s values based on its nearest neighbor but rather is a function of model covariates from the full sample, i.e., all clusters. We expect that on average a sufficient number of remaining urban clusters capture exclusive levels of the indicator of interest, especially since areas with higher heterogeneity (or diversity) such as cities, are more likely to be represented by a higher number of clusters. The extent of the ‘averaging’ is finally also tested via the cross-validation process. As such we can be assured that any model that passes the interpolation checks, produces final estimates that minimize the effects of averaged grids.

Despite the limitation of the ‘averaging’ effect, it is important to make the distinction that some ‘averaging’ at the 1km^2^ level is certainly no more erroneous, any probably less so, than greater ‘averaging’ at the national or first administrative level.

## Conclusions

The primary aim of our research has been in support of increasing vaccine uptake. To this end, we focused our work on developing hyperlocal estimates which could better inform local implementers and increase their ability to respond in a more targeted and impactful manner to community concerns and barriers. Our analytical objectives, as shared previously, were to (1) demonstrate the reliable estimation of attitudinal data at the hyperlocal level, and (2) better understand the loss of heterogeneity at higher levels of aggregation that are currently more common in making programmatic decisions.

The potential for mapping attitudinal data at the hyperlocal level via spatial interpolation is broad. Similar techniques could be used to understand health attitudes across many different public health concerns, particularly in low- and middle-income countries where reliable case data is scarcer. Metrics of health attitudes and behaviors that measure disease-related stigma, attitudes toward routine immunization and reproductive care, or perceived risk of illness at the hyperlocal level can provide insights that will fundamentally change how program planning decisions are made in a public health context.

Our research demonstrates that there is significant geographic heterogeneity across the determinants of COVID-19 vaccine hesitancy that is not captured by nationally representative survey data. By building on previous research producing hyperlocal estimates of demographic and socioeconomic indicators, we demonstrate the ability to also map attitudinal data down to one square kilometer. Using the WHO endorsed 3Cs framework of vaccine hesitancy, these attitudinal indicators allow us to better understand the unique determinants of vaccine hesitancy across an entire country at the neighborhood or community level. Given the difficulties of increasing COVID-19 vaccine uptake among hesitant populations, a better understanding of this variability will allow for more targeted communication and tailored interventions aimed at changing health attitudes and behaviors.

We see the strongest use cases for these data in local decision-making. National- or regional-level data could be used to make decisions regarding, for example, vaccine allocation at a state level. However, regional-level data does not help local program managers make decisions *within* their respective areas. As our data show, there are appreciable differences in each of the “3Cs” within many if not most states.

Our study contributes to existing research that finds recurring large, nationally representative, geotagged household surveys, such as the Nigeria DHS, can be used to reliably produce hyperlocal estimates of attitudinal data, which—up to this point, has not been done. We recognize the prevailing lack of available attitudinal data in such surveys and advocate for the inclusion of standardized survey questions pertaining to vaccine hesitancy in future rounds. Recognizing practicalities around survey length and country context, the most recently published WHO Behavioral and Social Drivers of Vaccination (BeSD) guidance has standardized a smaller number of priority questions and indicators that would serve this purpose well [34]. This BeSD guidance already delineates between priority, main, and optional survey questions for both childhood and COVID-19 vaccination so afford both flexibility and comparability across geographies and timelines. At the time of our research, the BeSD guidance was still being finalized.

Finally, we see opportunity for the use of hyperlocal attitudinal data in other public health contexts as well, aside from vaccine uptake support during the COVID-19 pandemic. Perceptions of access to medicine, as well as acceptance of ever-evolving medical advice and practices, may contribute significantly to the success or failure of other public health programs supporting populations with other communicable and non-communicable diseases. These hyperlocal data can support programmatic decision-making at a local level - where public health practitioners often work - not just national or regional levels, so that health program managers can be increasingly precise in their program implementations. We hope that improved availability of more granular data can be used to support public health programming worldwide.

### Supplementary Information

**Additional file 1:** A detailed explanation of the spatial interpolation process. An explanation of Fraym’s interpolation process is detailed further in Appendix A. A summary explanation of the interpolation process used by Fraym can also be found within the article by Brugh et al. coauthored by individuals also employed by Fraym. Some of these authors supported this research and served as reviewers to this article and are highlighted under Acknowledgements. The authors felt the interpolation process is a critical piece of context for this study, but not the subject of the study, so included this explanation as additional data file 1 rather than include it within the body of the article or rely solely on a reference to the previous publication. Sources [35] and [36] are only referred to in Appendix A, not within the manuscript.

**Additional file 2:** Benefits of the 3Cs model and indicator selection rationale. Our rationale for choosing the indicators and questions included in this study, as well as for aligning them to the 3Cs model, is detailed further in Appendix B. The authors believe this rationale is important to include, but not essential for and potentially distracting from understanding the study Methods section, so included this rationale as additional data file 2. Source [37] is only referred to in Appendix B, not within the manuscript.

## Data Availability

The 2018 Nigeria Demographic and Health Survey is available via the DHS Program website, https://dhsprogram.com/data/dataset/Nigeria_Standard-DHS_2018.cfm. The market research survey data collected in February 2022 are not publicly available but are available from the corresponding author on reasonable request. The hyperlocal estimates produced across the ten different countries are not publicly available but are being made available to authorized users through a password protected web-based data visualization dashboard. To receive access, users must be, or be directly supporting, verified public health stakeholders working towards increasing COVID-19 vaccine uptake in one of the ten countries.

## Abbreviations

1km^2^: One square kilometer
BeSD: Behavioral and Social Drivers of Vaccination
3Cs: Confidence, complacency, convenience
COVID-19: Coronavirus disease 2019
CATI: Computer assisted telephone interviewing
DHS: Demographic and Health Survey
IPF: Iterative proportional fitting
J&J GPH: Johnson and Johnson’s Global Public Health
LMICs: Low- and middle-income Countries
MoH: Ministry of Health
MCA: Multiple correspondence analysis
ML: Machine learning
PCA: Principal component analysis
PEPFAR: The United States President’s Emergency Plan for AIDS Relief
SAGE: Strategic Advisory Group of Experts on Immunization
WHO: World Health Organization

## Footnotes

1 A majority proportion is defined here as the top 3 quintiles, or top 60%, of data.

2 The minimum threshold for a majority proportion of low confidence at the LGA level was identified as 27 percent.

3 The minimum threshold for a majority proportion of smartphone owners at the LGA level was identified as 85 percent.

4 The minimum threshold for a majority proportion of Facebook usership at the LGA level was identified as 60 percent.
