# Supplementary material for: A novel adaptation of spatial interpolation methods to map health attitudes related to COVID-19
Source: BMC Proc. 2023 Jul 17;17(Suppl 7):17. doi: 10.1186/s12919-023-00264-z (PMC10351110; doi:10.1186/s12919-023-00264-z)
Supplement: Supplementary file 1 — Additional file 1: A detailed explanation of the spatial interpolation process. An explanation of Fraym’s interpolation process is detailed further in Appendix A. A summary explanation of the interpolation process used by Fraym can also be found within the article by Brugh et al. coauthored by individuals also employed by Fraym. Some of these authors supported this research and served as reviewers to this article and are highlighted under Acknowledgements. The authors felt the interpolation process is a critical piece of context for this study, but not the subject of the study, so included this explanation as additional data file 1 rather than include it within the body of the article or rely solely on a reference to the previous publication. Sources [35] and [36] are only referred to in Appendix A, not within the manuscript. [file 12919_2023_264_MOESM1_ESM.zip › Appendix A.pdf]

Appendix A

A detailed explanation of the spatial interpolation process

Fraym has built a patent-pending AI/ML software that weaves together high-quality household survey data with satellite imagery to create estimates of localized population characteristics at a $1km^2$ resolution. Fraym's AI/ML software uses geotagged survey responses, along with satellite imagery layers, to predict the percentage of respondents who exhibit the given characteristic within each $1km^2$ grid cell.

The first data input of the localized data modeling process is primary data from geo-referenced household surveys, which Fraym oversaw in the Nigeria data described in this research. The second major data input is satellite imagery and remotely sensed data products, including earth observation (EO) data, gridded population information (e.g., human settlement mapping), proximity to physical locations (e.g., health clinics, ports, roads, etc.), and biophysical surfaces like soil characteristics. As with the survey data, Fraym data scientists ensure that the software only uses high-quality imagery and derivative inputs. The typical Fraym AI/ML model will incorporate over 200 distinct types of imagery and remotely sensed data inputs. Any missing values are filled by the mean of a 5x5 kilometer focal window, a more robust version of nearest neighbors [36].

To create spatial layers from household survey data, Fraym uses a model-stacking ML approach to predict a continuous surface of the indicator of interest at a $1km^2$ resolution. This methodology builds upon existing, tested methodologies for interpolation of spatial data [10]. In sum, the software creates a model that identifies correlations between the scientifically sampled survey data at enumeration clusters and the satellite imagery and remotely sensed layers from the same location. The resulting model is used to predict the survey data for all non-enumerated

areas. A similar approach was pioneered by USAID's Demographic and Health Surveys (DHS) program in 2015 and has since then been improved upon by Fraym and others [12].

Fraym's ML process involves generating predictions from a set of base-learner models upon which a super-learner model is trained [10]. By leveraging multiple base models, the software can improve final predictions across large geographies. Models are tuned and evaluated using industry-standard cross-validation techniques, and the predictive power of smaller data sets is increased through systems of boosting, bagging, and k-fold cross validation [37].

Grid cells with no survey data are predicted by applying a model using the parameters generated in the train and tune process. For every data layer, Fraym data scientists examine the standard model metrics such as R-squared and Root Mean Square Error (RMSE) to relay quality. Generally, data layers have very robust indicators of quality. For example, an RMSE value of 0.025 for a proportional question from the survey means that roughly the average error between the prediction and the held-out enumeration area data was 2.5%. For proportional variables, if RMSE is greater than 0.1, then data layers are not used in production because they are too low quality. Similar thresholds are applied to non-proportional variables. In addition, comparisons are made between the spatial surface and the survey data at the lowest administrative level for which the survey was designed to be representative (e.g., departments). This survey mean is compared against the implied mean of the surface when all grids are appropriately aggregated through population weighted zonal statistics.

Although the Fraym software is proprietary, it is only automating existing techniques using prevalent AI/ML models to produce very granular estimates more rapidly, with greater cost efficiencies, at scale. The overall process of interpolating attitudinal data at a hyperlocal level using a model-stacking ML approach with survey and satellite data inputs is wholly replicable

using the techniques outlined in the referenced literature. The primary software used for these processes are R and Python, which are open source.
