# Supplementary material for: A novel adaptation of spatial interpolation methods to map health attitudes related to COVID-19
Source: BMC Proc. 2023 Jul 17;17(Suppl 7):17. doi: 10.1186/s12919-023-00264-z (PMC10351110; doi:10.1186/s12919-023-00264-z)
Supplement: Supplementary file 2 — Additional file 2: Benefits of the 3Cs model and indicator selection rationale. Our rationale for choosing the indicators and questions included in this study, as well as for aligning them to the 3Cs model, is detailed further in Appendix B. The authors believe this rationale is important to include, but not essential for and potentially distracting from understanding the study Methods section, so included this rationale as additional data file 2. Source [37] is only referred to in Appendix B, not within the manuscript. [file 12919_2023_264_MOESM2_ESM.zip › Appendix B.pdf]

Appendix B

Benefits of the 3Cs model and indicator selection rationale

Since WHO guidance on the 3Cs model available at the time did not specify the exact indicators and questions to use for COVID-19 vaccine hesitancy, we used the accompanying WHO SAGE Working Group Determinants of Vaccine Hesitancy Matrix (Determinants Matrix) and separate WHO Background Paper on Behavioral and Social Drivers of Vaccine Uptake (BeSD) to select them [4, 35, 38]. The Determinants Matrix categories include contextual influences, individual and group influences, and vaccine/vaccination-specific issues while the BeSD themes include thinking and feeling, motivation, social processes, and practical issues. With both frameworks, determinants/drivers could be readily mapped to the 3Cs model.

For example, the Confidence Index indicators listed in Table 1 are analogous to both 1) Determinants Matrix indicators “risk/benefit (perceived, heuristic)” and “immunisation as a social norm vs. not needed/harmful” under Individual and Group Influences and “risk/benefit (epidemiological and scientific evidence)” under Vaccine/Vaccination Specific Issues as well as 2) BeSD indicators “confidence in vaccine benefits” and “confidence in vaccine safety” under Thinking and Feeling and “peer norms” under Social Processes. While both resources were useful in determining indicators to include, we agreed with the WHO SAGE Working Group on Vaccine Hesitancy that the 3Cs model is “intuitive and thus the easiest to grasp.”

As such, we decided that the 3Cs model would have the greatest utility and interpretability for our purposes because A) the objectives of this research were to determine 1) whether attitudinal data could be interpolated and 2) whether there was significant hyperlocal heterogeneity within attitudes related to vaccine hesitancy and B) outputs from this research were intended to be used by public health stakeholders to improve vaccine uptake. While all three options (3Cs

model, determinants matrix, BeSD) were sufficient to meet our research objectives, the 3Cs model was perceived as more likely to be understood and used by a wide variety of public health stakeholders.
